# Developing an algorithm to identify people with Chronic Obstructive Pulmonary Disease (COPD) using administrative data

**DOI:** 10.1186/1472-6947-12-38

**Published:** 2012-05-22

**Authors:** Margrethe Smidth, Ineta Sokolowski, Lone Kærsvang, Peter Vedsted

**Affiliations:** 1 The Research Unit for General Practice Aarhus, Bartholins Allé 2, Aarhus University, 8000, Aarhus C, Denmark; 2 The Department of General Medicine Bartholins Allé 2, Aarhus University, 8000, Aarhus C, Denmark; 3 The Central Denmark Region, Skottenborg 26, 8800, Viborg, Denmark

## Abstract

**Background:**

An important prerequisite for the Chronic Care Model is to be able to identify, in a valid, simple and inexpensive way, the population with a chronic condition that needs proactive and planned care. We investigated if a set of administrative data could be used to identify patients with Chronic Obstructive Pulmonary Disease in a Danish population.

**Methods:**

Seven general practices were asked to identify patients with known Chronic Obstructive Pulmonary Disease in their practices. For the 266 patients (population A), we used administrative data on hospital admissions for lung-related diagnoses, redeemed prescriptions for lung-diseases drugs and lung- function tests combined to develop an algorithm that identified the highest proportion of patients with Chronic Obstructive Pulmonary Disease with the fewest criteria involved. We tested nine different algorithms combining two to four criteria. The simplest algorithm with highest positive predictive value identified 532 patients (population B); with possible diagnosis of Chronic Obstructive Pulmonary Disease in five general practices. The doctors were asked to confirm the diagnosis. The same algorithm identified 2,895 patients whom were asked to confirm their diagnosis (population C).

**Results:**

In population A the chosen algorithm had a positive predictive value of 72.2 % and three criteria: a) discharged patients with a chronic lung-disease diagnosis at least once during the preceding 5 years; or b) redeemed prescription of lung-medication at least twice during the preceding 12 months; or c) at least two spirometries performed at different dates during the preceding 12 months. In population B the positive predictive value was 65.0 % [60.8;69.1 %] and the sensitivity 44.8 % [41.3;48.4 %)] when the “uncertain” were added to where doctors agreed with the diagnosis. For the 1,984 respondents in population C, the positive predictive value was 72.9 % [70.8;74.8 %] and the sensitivity 29.7 % [28.4;31.0 %].

**Conclusions:**

An algorithm based on administrative data has been developed and validated with sufficient positive predictive value to be used as a tool for identifying patients with Chronic Obstructive Pulmonary Disease. Some of the identified patients had other chronic lung-diseases (asthma). The algorithm should mostly be regarded as a tool for identifying chronic lung-disease and further development of the algorithm is needed.

**Trial registration:**

www.clinicaltrials.gov (NCT01228708)

## Background

The group of people living with non-communicable diseases (NCD) or chronic conditions is growing as life-expectancy increases, treatment options improve, inappropriate lifestyle spreads, and the diagnostic activity grows. Thus, we need initiatives to devise an efficient healthcare strategy [1]. To offer a professional and effective treatment, the care needs to be well structured and integrated within the healthsystem [2,3]. One model which frames comprehensive care is the Chronic Care Model [4]. Identifying patients already treated in the healthsystem seems beneficial for planning their care and might diminish the number of emergency contacts they have with the healthsystem. The model requires that the population with the chronic conditions and the initial stages of disease is identified [5]; this prerequisites a comprehensive, timely and valid registration in all parts of a healthcare system.

In a Dutch study, Steuten et al. found that self-reported medication compliance, physical activity, disease specific knowledge, non-smoking behaviour, patient satisfaction and health related quality of life increased in populations with chronic disease when implementing a disease management program focusing on patient education, protocolised assessment and treatment of COPD, and care coordination. They also found improvement in health utility [6].

Chronic Obstructive Pulmonary Disease (COPD) is an under-diagnosed, irreversible and potentially life-threatening condition where secondary prevention, treatment and rehabilitation can help control the symptoms, increase the patient’s quality of life and delay disease progression [7]. Newly published results indicate that of 3 million people at 35 years or older, 430,000 (14.3 %) live with COPD [8] in Denmark; and of these approximately 120,000 (4.0 %) have been diagnosed [9]. One very important prerequisite for implementing high impact disease management programs is being able to identify the target population [10]. In Denmark, patients in hospitals are coded according to the International Classification of Diagnosis (ICD-10) which also identifies patients with chronic conditions. However, most people with chronic diseases are seen in primary care, and in Denmark a systematic coding of diagnoses has not yet been fully established in general practice. Consequently, we need to develop additional models for identification based on administrative data Therefore, the aim of this study is to develop and test a method for identification of patients living with COPD in a Danish population based on administrative data.

## Methods

### Setting

The use of healthcare services is free at the point of use in Denmark, and the healthcare system is financed by taxes. The approximately 3,600 general practitioners (GPs) have an average of 1500 patients on their list as 98 % of the population is registered with a GP. The GPs are gatekeepers in terms of access to the rest of the healthcare system; a referral from the GP is mandatory except for emergency admission. All GPs use electronic medical records which mean that each patient’s record is electronically searchable and all communication in and out of practice is electronically based.

### Overall study design

The algorithm was developed in three steps and in three different GP and patient populations. First, one group of GPs were asked to identify a list of their patients with a known diagnosis of COPD -population A . Based on administrative data, the simplest algorithm which identified as many of these as possible was developed. Then patients with COPD were sampled according to the algorithm and GPs in other practices were asked to verify the diagnosis from their records -population B. Finally, another group of sampled patients were asked to verify whether they had COPD or not - population C.

### Register data

All citizens living in Denmark are registered with a unique personal identification number called a CPR-number. Linking of all national registries with the CPR-number is therefore possible at the individual level [11]. Data about inhabitants’ use of general practice was obtained from the Danish National Health Insurance Service Registry (see Additional file 1 for healthcare use data) [12]. From the Regional Prescription Registry which contains information on all dispensed prescriptions (ATC-codes) in the Central Denmark Region (see Appendix 1 for ATC-codes used) [13] data on sampled inhabitants’ use of prescribed medication was collected. Out-patient visits, emergency-room visits, hospital admissions and discharges were collected from the Patient Administrative System (PAS) based on ICP-10 codes (see Additional file 1 for diagnosis).

### Algorithm development

Seven practices with 26 GPs in the county of Aarhus accepted to identify all patients in their practice with a known diagnosis of COPD. For these patients - population A, register data was collected based on their CPR-number on in- and outpatient attendance five years back, redeemed prescriptions during the preceding 12 months, and the number of spirometries performed at GPs and specialists during the preceding 12 months (See Additional file 2). Different combinations were then set up and tested for performance in identifying the patients with COPD identified by the GPs. The algorithm with the highest positive predictive value (PPV), and which was also the simplest, was regarded as being most appropriate. Thus, each algorithm was tested for the ability to identify the patients identified with COPD measured by PPV with 95 % confidence intervals (95%CI). A total of nine relevant algorithms were evaluated (See Additional file 3).

### Algorithm testing

#### GP verification

The chosen algorithm was used to identify patients with possible COPD in five different practices with a total of 17 GPs in the Central Denmark Region - population B. The GPs were asked to confirm or repudiate the diagnosis of COPD or state “unsure”. The GPs were asked to refer to the GOLD guidelines where the diagnosis of COPD is confirmed by means of post-bronchodilator spirometry which shows a forced expiratory volume in one second (FEV1)/forced vital capacity (FVC) < 70 %[14]. The algorithm identified patients who had been in contact with different parts of the healthcare system for lung-related complaints; consequently the category “unsure” was added to the confirmed verified diagnosis of COPD.

### Patient verification

Patients sampled by means of the algorithm were asked to verify their diagnosis. As part of a large randomised study, a questionnaire was sent to patients identified by the algorithm in the Ringkoebing-Skjern and Ikast-Brande municipalities - population C. These patients were asked to confirm or repudiate their potential COPD diagnosis. Non-responders were sent a reminder after three weeks.

### Analysis

For all algorithms, sensitivity, specificity as well as positive and negative predictive values was calculated, and for the final algorithm, it was calculated for age groups too. Hansen et al. found a 9 % overall prevalence for COPD in the population aged 45–85 and this was used as the prevalence measure in order to identify patients with COPD already being treated in the healthcare system. Based on the prevalence, age specific PPV was calculated [15]. For patients aged 35–44, the prevalence for the 45–54 year olds was used and for patients aged 85 and above the prevalence for patients aged 75–84 years was used.

In connection with patient validation, responders and non-responders were compared in terms of gender, age and the criteria used in the algorithm using two sample *t*-test. The distribution of gender within the two groups was tested by Pearson's chi-squared test and Fischer’s exact test was used for the comparison of the criteria used to identify the patients. Analyses were performed using STATA version 11.0. (StataCorp, College Station, Texas).

### Ethics

The study was approved by the Danish Data Protection Agency (J.nr. 2008-41-2855), the Danish National Board of Health (J. NR.: 7-604-04-2/71/EHE) and the RCT indexed at http://www.clinicaltrials.gov (NCT01228708).

## Results

### Algorithm development

The GPs identified 266 patients in their practices with COPD - population A. Of the algorithms tested on these patients, the simplest algorithm with the highest PPV contained three criteria and a PPV of 72.2 %. The nine best performing algorithms can be seen in Additional file 3.

### Algorithm testing

#### GP verification - population B

**Figure 1:**
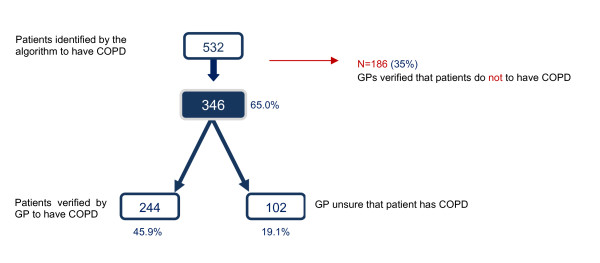
**Patients identified by algorithm and verified by GPs - population B.** Flowchart showing the number of patients for which GPs can verify the diagnosis of COPD when the patients have been identified by the COPD algorithm developed in the study.

**Figure 2:**
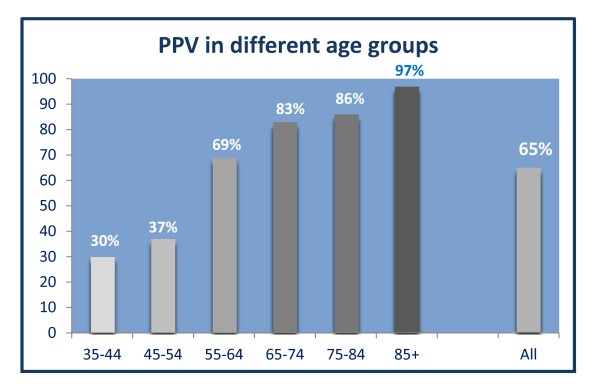
**Positive Predictive Values when patients verified by GPs in population B.** The Positive Predictive Value when GPs verified the COPD diagnosis for patients identified by the algorithm developed in the study. The patients were divided into ten-year age groups.

The second group of GPs verified that 244 (45.9 %) of the algorithm identified patients had COPD. For 102 (19.1 %) patients, the GPs were not sure of the diagnoses (Figure 1). Thus, the PPV for a possible or definite COPD was 64.8 % and the sensitivity vas 44.8 [95%CI: 41.3-48.4] and the specificity 97.7 % [95 % CI: 97.3-98.0] (Se Additional file 4). The 10-year age span specific PPVs varied from 30-97 % (Figure 2).

### Patient verification - population C

**Figure 3:**
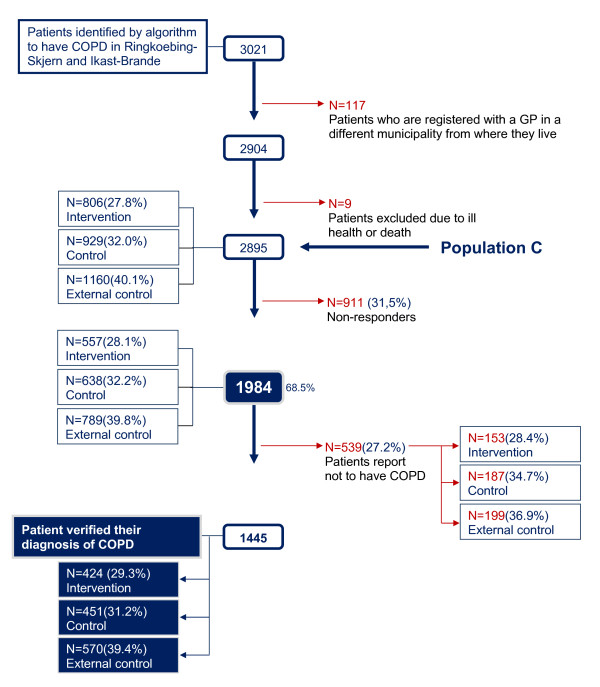
**Patients who verified their COPD diagnosis - population C.** Flowchart showing the number of patients who verified their COPD diagnosis when identified by the algorithm developed in the study.

**Figure 4:**
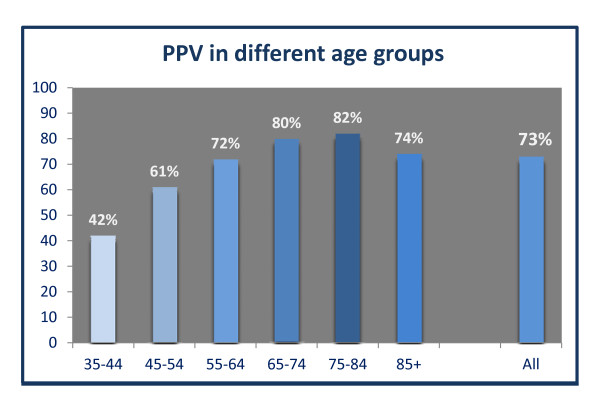
**The Positive Predictive Value when patients verified their COPD diagnosis in population C.** The patients were identified by the algorithm developed in the study and divided into ten-year age groups.

A total of 1,984 (68.5 %) patients identified by the algorithm and eligible for inclusion in the study returned the questionnaire (Figure 3). The sensitivity was 29.7 % [95 % CI: 28.4-31.0] and the specificity 98.9 % [95 % CI: 98.8-99.0] and the overall PPV was 72.8 % [95%CI: 70.8 %;74.8 %] with a prevalence of COPD of 9 % in the Danish population (See Additional file 5). The 10-year age span specific PPVs ranged from 41.8-81.8 % (Figure 4).

No statistically significant difference was found between responders and non-responders regarding gender (p = 0.061) where 54.8 % of responders and 53.7 % of non-responders were women. On averages, responders were 4.5 years older than non-responders, 65.6 (SD 12.6) years and 61.1 (SD 14.8) respectively, (p < 0.001). Of the identified patients, 80.2 % were found using one criterion - 78.5 % in the responders and 81.9 % in the non-responders group. More responders (121 (6.1 %)) than non-responders (36 (3.9 %)) were identified by their use of medication and having had a spirometry performed. All three criteria in the algorithm identified three (0.3 %) of the non-responders and 27 (1.4 %) of the responders, (p = 0.005).

## Discussion

### Main findings

Developing an algorithm for COPD combining data from the GP, administrative data and verifying the performance of this algorithm among GPs and patients, showed that an algorithm using three variables derived from register data could identify between 30 % and 97 % depending on age of the COPD population. The relatively low PPV for younger groups might be explained by the inclusion of many patients with asthma.

### Algorithm performance

The chosen algorithm identified close to three quarters of patients with COPD in the population aged 35 years and up in population B and C. If we look at the population aged 55 years and up the identification rate increased. Age was therefore an important variable that could be considered in the algorithm as well.

### Strengths and weaknesses

We developed the algorithm from registry data on hospital and prescription data which have been shown to be highly valid [16-19]. When testing the algorithm with patients in population C, the percentages verifying their diagnosis were higher than testing it with GPs in population B.

We have a selection bias as there is a higher likelihood of responding when one is identified by three criteria instead of two or one. This leads us to believe that more patients in population C with an already verified diagnosis have answered the survey. The older the age group, the less the difference was in the PPV between the patients' verification of their diagnosis in population C and the GPs' verification in population B. The responders in population C were representative for the population identified by the algorithm in terms of gender.

A very high specificity of the algorithm is one of its strengths. It ensures that people who have been hospitalized with a lung-related diagnosis, been prescribed lung-related medication or had a spirometry test for other diagnoses will not unnecessarily be suspected of having COPD.

When asking the initial 26 GPs to identify the patients with COPD in their practice - population A, the GOLD guidelines for COPD was not specified [20]. This could both mean that less and that more patients could have been identified, probably more, considering the fact that it has been suggested that the overall prevalence of people with COPD is 14.2 %.This number includes a sizeable portion of people with mild COPD who would not have come in contact with the healthcare system regarding their lung-disease. Thus; we might regard the patients identified by the algorithm as being the sickest patients.We decided to use the overall prevalence of 9 % as suggested by Hansen et al. They standardized to the Danish population based on their study of a stratified sample of 4,757 people out of 299,000 Danes aged 45–84 years. The 14.3 % prevalence suggested by Loekke et al. only applied to people aged 35 and above and was calculated on the basis of a study of a much smaller sample. We therefore found it relevant to use the prevalence from the study by Hansen et al. in our study.

We chose to consider the ‘unsure’ answers as a positive diagnoses as the patients in population B had been identified by at least one of the criteria and therefore had been in contact with the healthcare system due to a lung-related issue. In addition, the GPs could not reject the diagnosis. An information bias is that not all the ‘unsure’ answers from GPs might be positive answers to a COPD diagnosis, but could be identifying another lung-related disease in population B. We could consider that the algorithm identifies patients with a lung-related disease and not only patients with COPD.

If we want to further develop the algorithm to only identify patients with COPD, we could include more variables, e.g. the International Classification of Primary Care (ICPC-2) codes, the use of which will become compulsory in general practice in Denmark before 2013. We could decide to use the algorithm in its present form for people aged 55 or older as it has the best properties in this group.

### Comparisons with other studies

Different studies have used data from different sources to identify people who have or might have COPD. In the United States, a study developed an algorithm for identifying people at risk of having unidentified COPD from healthcare utilization data and found it to be a useful screening tool. Another American study found that pharmacy utilization efficiently identified persons at risk of undiagnosed COPD [21]. A Canadian study used health administrative data to identify cohorts of patients with asthma with the purpose of monitoring the population and for healthcare research [22]. They found that their algorithm would be enhanced if hospital data was added to the GPs' data. These studies support our approach in this study of being able to identify patients with COPD from administrative data in order to enhance the proactive care and have an identified population for research and quality improvement purposes.

A Danish study suggests that COPD is under-recorded when patients have been admitted with a different acute lung-related diagnosis [23]. This emphasises the need to use the diagnosis for all lung-related diseases when identifying patients.

An English study found that when general practice had identified a high-risk COPD population from their own data, a trend towards reduction of hospital admissions and inpatient days was found compared to the situation where the COPD population was not identified [24]. This could be investigated further in a Danish setting by using the algorithm to identify a Danish population.

As COPD is one of the chronic conditions, the Danish health system wishes to identify, monitor and enhance the rehabilitation for [25], a targeted system that efficiently identifies patients with COPD is attractive from the perspective of secondary prevention which serves to identify and treat people with preclinical disease and those who have developed the disease. Organized efforts aimed at identification for proactive care of the patients with COPD are especially important as the health gains are better the earlier the patient is diagnosed, and the long-term prognosis of COPD may improve with early intervention [26,27].

## Conclusions

We have developed an algorithm based on administrative data and we have tested it with both GPs and patients. The algorithm is found to have sufficient PPV to be used as a screening tool in the identification of patients with COPD in a general population in Denmark, especially among the group of elderly. This algorithm can be useful for the health system both to identify the patients who will benefit from proactive care and those who need integrated care for this progressive and irreversible illness.

## Abbreviations

CI: Confidence Interval; COPD: Chronic Obstructive Pulmonary Disease; GP: General Practitioner; ICD-10: International Classification of Disease - version 10; ICPC-2: International Classification of Primary Care - version 2; NPV: Negative Predictive Value; PAS: Patient Administrative System; PPV: Positive Predictive Value; SD: Standard Deviation.

## Competing interests

The authors declare that they have no competing interests.

## Authors’ contributions

PV conceived the study idea, participated in its design and in developing the algorithm and he helped to draft the manuscript. IS participated in the study design and in developing the algorithm and performed the statistical analyses. LK carried out data collection related to the GPs' verification of the algorithm. MS coordinated the study, carried out the questionnaire survey, participated in the study design and drafted the manuscript. All authors have read and approved the final manuscript.

## Pre-publication history

The pre-publication history for this paper can be accessed here:

http://www.biomedcentral.com/1472-6947/12/38/prepub

## Supplementary Material

Additional file 1 The conditions for identification by the algorithm developed and validated in the study. Click here for file

Additional file 2 Descriptive data for patients identified by their GP to have COPD - population A. Click here for file

Additional file 3 Patient identified with COPD using different algorithms for population A. An inclusion prerequisite was to be aged 35 or above and alive at the time of identification in the registries. Click here for file

Additional file 4 The characteristics of algorithm-identified patients for whom the GP were asked to verify the COPD diagnosis - population B. The prevalence of COPD suggested by Hansen et al.(16)was used. Click here for file

Additional file 5 The characteristics of algorithm-identified patients whom were asked to verify their COPD diagnosis - population C. The patients were divided into ten year age groups. Data from Statistics Denmark were used for the population in 2007. The prevalence of COPD suggested by Hansen et al.(16)was used. Click here for file
