# Supplementary material for: Developing an algorithm to identify people with Chronic Obstructive Pulmonary Disease (COPD) using administrative data
Source: BMC Med Inform Decis Mak. 2012 May 22;12:38. doi: 10.1186/1472-6947-12-38 (PMC3444358; doi:10.1186/1472-6947-12-38)
Supplement: Additional file 1 — The conditions for identification by the algorithm developed and validated in the study. [file 1472-6947-12-38-S1.pdf]

ALGORITHM:
Age: All citizens ages 35 and up at the time of identification in the registry.
Vital status: Alive at the time of identification in the registry.
Selection of COPD patients: People who meet at least one of the following criteria can be sampled as possible patients with COPD:

1. Hospital contact. People who have been hospitalised or have had an outpatient visit at least once during the last 5 years with one or more of the following ICD-10 codes as main diagnosis:

Bronchitis without specification	DJ40
Bronchitis without specification	DJ409
Simple and mucopurulent chronic bronchitis	DJ41
Bronchitis chronica simplex	DJ410
Bronchitis chronica mucopurulenta	DJ411
Bronchitis chronica simplex et mucopurulenta, mixed type	DJ418
Chronic bronchitis without specification	DJ42
Chronic bronchitis without specification	DJ429
Tracheobronchitis chronica	DJ429A
Tracheitis chronica	DJ429B
Expansion of the lungs	DJ43
MacLeod's syndrome	DJ430
Emphysema pulmonum unilaterale	DJ430A
Emphysema pulmonum panlobulare	DJ431
Emphysema pulmonum panacinare	DJ431A
Emphysema pulmonum centrilobulare	DJ432
Emphysema, other kinds	DJ438
Emphysema without specification	DJ439
Emphysema pulmonum bullosum	DJ439A
Chronic obstructive lung disease, other	DJ44
Chronic obstructive lung disease, with acute lower respiratory tract infection	DJ440
Chronic obstructive lung disease, with acute exacerbation without specification	DJ441
Chronic obstructive lung disease, other specified version	DJ448
Bronchitis chronica obstructive	DJ448A
Bronchitis chronica asthmatica	DJ448B
Chronic obstructive lung disease without specification	DJ449
Expansion of bronchia	DJ47
Bronchiectasia	DJ479
Respiratory insufficiency not classified any other place	DJ96
Insufficientia respiratoria acuta	DJ960
Insufficientia respiratoria chronic	DJ961
Respiratory insufficiency without specification	DJ969

Only the diagnosis coding was examined, not the way the patients have been referred or any other matters.

2. COPD medicine. People who within the last 12 months from the time of identification from the register have redeemed at least 2 prescriptions on different dates with one or more, of the following ATC codes: R03AC; R03AK; R03BA; R03BB; R03CC; R03DA; R03DC; V03AN01

3. Spirometry in general practice. People who, within the last 12 months from the time of identification in the register, have had at least 2 spirometry tests on different dates and defined as a fee for lab test called:
7113 Expanded lung function test verified by spirometry or
7121 Double lung function test for exertion provoked asthma or reversibility test done by spirometry in the same consultation.
