# Supplementary material for: Developing an algorithm to identify people with Chronic Obstructive Pulmonary Disease (COPD) using administrative data
Source: BMC Med Inform Decis Mak. 2012 May 22;12:38. doi: 10.1186/1472-6947-12-38 (PMC3444358; doi:10.1186/1472-6947-12-38)
Supplement: Additional file 2 — Descriptive data for patients identified by their GP to have COPD - population A. [file 1472-6947-12-38-S2.pdf]

N=226		Patients with COPD N	%		Accumulated number of patients N	%
Number of admissions with a lung related diagnosis						
	01.04.07-31.03.08	48	18.0	Period 1	11	4.1
	01.04.06-31.03.07	42	15.8	Period 1 or 2	57	21.4
	01.04.05-31.03.06	37	13.9	Period 1,2 or 3	78	29.3
	01.04.04-31.03.05	32	12.0	Period 1,2,3 or 4	91	34.2
	01.04.03-31.03.04	34	12.8	Period 1,2,3,4 or 5	100	37.6
Redeemed prescription medication for a lung related diagnosis	01.04.07-31.03.08					
	At least once	188	70.7			
	At least twice	176	70.7			
Spirometry performed at GP	01.04.07-31.03.08	92	34.6			
Reversibility test performed at GP	01.04.07-31.03.08	19	7.1			
1 x redeemed prescription and spirometry / reversibility test at GP		77	28.9			
2 x redeemed prescription and Spirometry / reversibility test at GP		75	28.2			
Spirometry performed at Consultant	01.04.07-31.03.08					
	At least once	2	0.8			
	At least twice	0	0.0			
