# Supplementary material for: Developing an algorithm to identify people with Chronic Obstructive Pulmonary Disease (COPD) using administrative data
Source: BMC Med Inform Decis Mak. 2012 May 22;12:38. doi: 10.1186/1472-6947-12-38 (PMC3444358; doi:10.1186/1472-6947-12-38)
Supplement: Additional file 3 — Patient identified with COPD using different algorithms for population A. An inclusion prerequisite was to be aged 35 or above and alive at the time of identification in the registries. [file 1472-6947-12-38-S3.pdf]

Algorithm	Identified All	%
**1.**<br>Inpatient at least once during the last 5 years<br>Redeemed prescription medication at least twice during the last year<br>Redeemed prescription medication once and spirometry performed once at GP during the last year	189	71.1
**2.**<br>Inpatient at least once during the last 5 years<br>Redeemed prescription medication at least twice during the last year<br>Redeemed prescription medication once and spirometry performed once at GP or consultant during the last year	189	71.1
**3.**<br>Inpatient at least once during the last 5 years<br>Redeemed prescription medication at least twice during the last year<br>Spirometry performed at least twice during the last year at GP<br>Redeemed prescription medication once and spirometry performed once at GP or consultant during the last year	193	72.6
**4.**<br>Inpatient at least once during the last 5 years<br>Redeemed prescription medication at least twice during the last year<br>Spirometry performed at least twice during the last year at GP<br>Redeemed prescription medication once and spirometry performed once at GP or consultant during the last year	189	71.1
**5.**<br>Inpatient at least once during the last 4 years<br>Redeemed prescription medication at least twice during the last year<br>Spirometry performed at least twice during the last year at GP<br>Redeemed prescription medication once and spirometry performed once at GP or consultant during last year	192	71.4
**6.**<br>Inpatient at least once during the last 3 years<br>Redeemed prescription medication at least twice during the last year<br>Spirometry performed at least twice at GP during the last year<br>Redeemed prescription medication once and spirometry once at GP or consultant during the last year	191	71.8
**7.**<br>Inpatient at least once during the last 2 years<br>Redeemed medication at least twice during the last year<br>Spirometry performed at GP at least twice during the last year<br>Redeemed prescription medication once and spirometry done once at GP or consultant during the last year	190	71.4
**8.**<br>Inpatient at least once during the last 5 years<br>Redeemed prescription medication at least twice during the last year	188	70.7
**9.**<br>**Inpatient at least once during the last 5 years**<br>**Redeemed prescription medication at least twice during the last year**<br>**Spirometry performed at least twice at different dates during the last year at GP**	**192**	**72.2**
