# Supplementary material for: Developing an algorithm to identify people with Chronic Obstructive Pulmonary Disease (COPD) using administrative data
Source: BMC Med Inform Decis Mak. 2012 May 22;12:38. doi: 10.1186/1472-6947-12-38 (PMC3444358; doi:10.1186/1472-6947-12-38)
Supplement: Additional file 4 — The characteristics of algorithm-identified patients for whom the GP were asked to verify the COPD diagnosis - population B. The prevalence of COPD suggested by Hansen et al.(16)was used. [file 1472-6947-12-38-S4.pdf]

GP	N	n	Algorithm identified COPD		GP verified COPD		Sensitivity (%) [95% CI]	Specificity (%) [95% CI]	PPV (%)[95% CI]	NPV (%) [95% CI]	Prevalence (%)[95% CI]
	Practice population	Expected practice population COPD PPV=9%	Yes + uncertain	None	Yes + uncertain	None					
**1**	2,821	254	153	2,668	118	35	46.5 [40.2-52.8]	98.6 [98.1-99.0]	77.1 [69.7-83.5]	94.9 [94.0-95.7]	9.0 [8.0-10.1]
**2**	2,124	191	152	1,972	78	74	40.8 [33.8-48.2]	96.2 [95.2-97.0]	51.3 [43.0-59.5]	94.3 [93.2-95.3]	9.0 [7.8-10.3]
**3**	1,636	147	81	1,555	45	36	30.6 [23.3-38.7]	97.6 [96.7-98.3]	55.6 [44.1-66.6]	93.4 [92.1-94.6]	9.0 [7.6-10.5]
**4**	877	79	66	811	49	17	62.0 [50.4-72.7]	99.9 [96.6-98.8]	74.2 [62.0-84.2]	96.3 [94.8-97.5]	9.0 [7.2-11.1]
**5**	1,117	101	80	1,037	56	24	55.5 [45.2-65.3]	97.6 [96.5-98.5]	70.0 [58.7-79.7]	95.7 [94.2-96.8]	9.0 [7.4-10.9]
**All**	8,575	772	532	8,043	346	186	44.8 [41.3-48.4]	97.6 [97.3-97.9]	65.0 [60.8-69.1]	94.7 [94.2-95.2]	9.0 [8.4-9.6]
