# Supplementary material for: Developing an algorithm to identify people with Chronic Obstructive Pulmonary Disease (COPD) using administrative data
Source: BMC Med Inform Decis Mak. 2012 May 22;12:38. doi: 10.1186/1472-6947-12-38 (PMC3444358; doi:10.1186/1472-6947-12-38)
Supplement: Additional file 5 — The characteristics of algorithm-identified patients whom were asked to verify their COPD diagnosis - population C. The patients were divided into ten year age groups. Data from Statistics Denmark were used for the population in 2007. The prevalence of COPD suggested by Hansen et al.(16)was used. [file 1472-6947-12-38-S5.pdf]

Age group	Sensitivity (%) [95% CI]	Specificity (%) [95% CI]	PPV (%)[95% CI]	NPV (%) [95% CI]	Prevalence (%)[95% CI]
**35-44**	7.78 [5.98-9.93]	99.0 [99.2-99.5]	41.8 [33.6-50.4]	94.5 [94.0-94.9]	5.90 [5.50-6.32]
**45-54**	20.7 [17.9-23.7]	99.2 [99.0-99.3]	60.5 [54.4-66.4]	95.2 [94.8-95.6]	5.90 [5.50-6.31]
**55-64**	25.7 [23.3-28.2]	98.9 [98.4-99.0]	71.8 [67.4-75.9]	92.1 [91.6-92.1]	10.2 [9.67-10.7]
**65-74**	29.5 [27.2-31.8]	98.3 [98.0-98.6]	80.0 [76.6-83.1]	86.0 [85.3-86.8]	18.4 [17.6-19.2]
**75-84**	24.8 [22.6-27.1]	97.7 [97.2-98.2]	81.6 [77.6-85.2]	76.5 [75.3-77.8]	28.5 [27.3-29.8]
**85+**	13.4 [10.6-16.6]	98.1 [97.2-98.8]	74.0 [64.0-82.4]	74.0 [71.9-76.0]	28.5 [26.5-30.6]
**All**	29.7 [28.4-31.0]	98.9 [98.8-99.0]	72.9 [70.8-74.8]	93.4 [93.2-93.6]	9.00 [8.76-9.24]
